# Cutaneous Metastatic Adenocarcinoma of the Colon to the Scalp

**DOI:** 10.14740/wjon862w

**Published:** 2015-02-14

**Authors:** Georgios P. Fragulidis, Antonios Vezakis, Michael K. Derpapas, Vassiliki Michalaki, Athanassios Tsagkas, Andreas A. Polydorou

**Affiliations:** a The 2nd Department of Surgery, Aretaieio Hospital, University of Athens Medical School, Greece; b Department of Oncology, Aretaieio Hospital, University of Athens Medical School, Greece; c Department of Pathology, Aretaieio Hospital, University of Athens Medical School, Greece

**Keywords:** Colorectal cancer, Scalp metastasis, Skin metastasis, Cutaneous metastasis

## Abstract

Cutaneous metastases from colorectal cancer are relatively uncommon presenting in fewer than 5% of patients but they are very important to recognize as they signify disseminated disease and poor prognosis. We describe a case a 62-year-old patient diagnosed with scalp metastasis during his systemic chemotherapy treatment for a colorectal carcinoma stage IVb who underwent excisional biopsy of the metastatic lesion. The identification of cutaneous metastases from colorectal cancer can radically alter therapeutic plans as they typically indicate a wide spread disease. Although they can be observed at any stage of malignancy, early recognition can lead to accurate and prompt diagnosis and timely treatment.

## Introduction

Colorectal adenocarcinoma most frequently metastasizes to the liver, the peritoneum, the pelvis, the lung and bone [1]. Cutaneous metastases of colorectal adenocarcinoma are rare with an incidence of 5% in these patients, while skin involvement is even rarer [2, 3].

They can be developed on average 16.5 to 30.8 months after a formal diagnosis of colorectal cancer and the primary sites for metastases have been reported as follows: rectum (55%), sigmoid colon (17%), transverse colon (9%), rectosigmoid (7%), cecum (4%), and ascending colon (4%) [4, 5]. Cutaneous involvement occurs mostly on the incision scars but is also seen on the skin of the extremities, head and neck, and penis by either lymphatic or hematogenous spread or by implantation during surgery [6]. This manifestation of remote metastasis from colorectal adenocarcinoma is more often detected in the settings of widespread disease. Thus, early recognition and diagnosis of the cutaneous metastatic lesion can severely alter the treatment and the prognosis of the disease [7].

Among cutaneous metastasis of colon cancer, scalp is one of the uncommon sites and has been reported in small case series and case reports [8-11]. We present a 62-year-old patient who developed a scalp metastasis during his palliative care follow-up for a stage IVb colorectal carcinoma.

## Case Report

**Figure 1:**
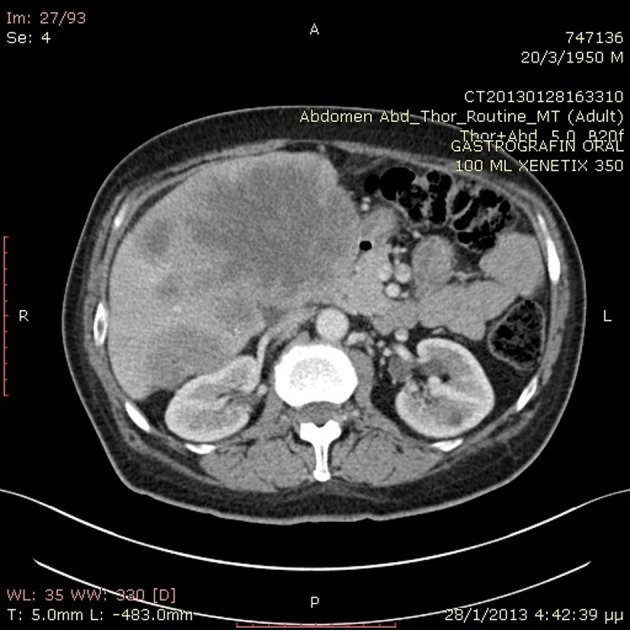
Abdominal CT scan revealed metastatic liver disease.

**Figure 2:**
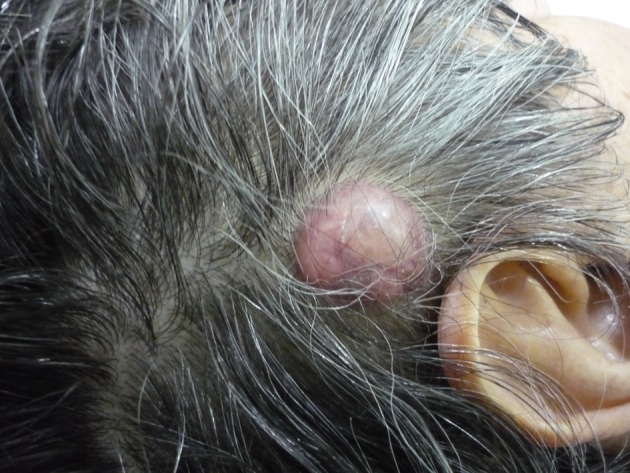
Gross photo of the metastatic lesion of the scalp.

**Figure 3:**
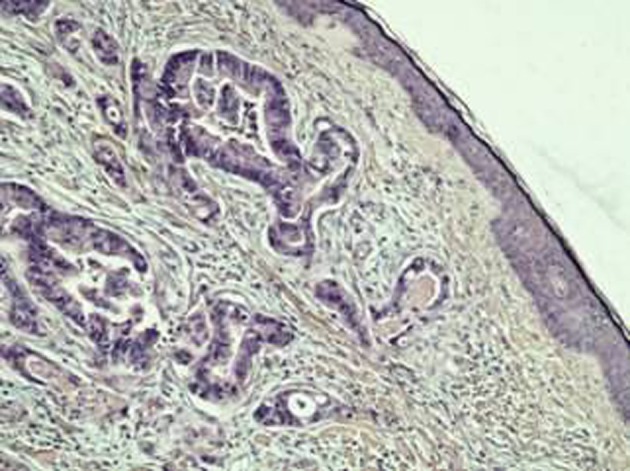
Histological section from skin specimen showing metastatic adenocarcinoma of a colon primary (H&E × 100).

A previously healthy 62-year-old Caucasian male was referred to our department with a 6-month history of diarrhea and weight loss. He underwent colonoscopy which revealed an invasive, hemorrhagic, ulcerated mass of the rectosigmoid area causing obstruction. Serum tumor markers were CEA > 1,500 ng/mL and CA19.9 > 1,200 U/mL. Histological examination of the tumor revealed a G2 grading adenocarcinoma of the colon. CT scans of the thorax and abdomen detected metastatic disease of the lungs and liver (Fig. 1) respectively (stage IVb). Hence, a self-expanding stainless steel stent was successfully endoscopically placed. The patient began systemic therapy with FOLFOX plus bevacizumab. Four months later the stent was obstructed from tumor ingrowth and a new stent was placed (stent to stent) while a subsequent CT scan confirmed progression of disease. At that time, patient presented a cystic lesion of the scalp at the right temporal area (Fig. 2). Histological analysis of the excised lesion of the scalp revealed a metastatic medially differentiated adenocarcinoma, most likely originating from the large bowel, which infiltrated the subcutaneous tissue and the skin (Fig. 3). A palliative care was followed as he had rapid physical decline and he was discharged home under a nursing care where he died 2 weeks later.

## Discussion

Cutaneous metastasis in cancer patients with internal malignancies can be diagnosed at the time of the initial presentation of the primary and during the clinical course. More often, it can be an indicator of cancer recurrence as they can be developed in more than 10 years after the initial cancer diagnosis in approximately 9% of patients. In patients with no evidence of primaries, cutaneous metastasis is most commonly seen as first sign in lung, kidney and ovary malignancies [12].

Data from the two pivotal studies conducted by Lookingbill et al showed that the frequency of skin involvement in cancer patients with widespread disease was 9.6%. Cutaneous metastases were present in 1.3% of patients at the time of identification of the primary. In colorectal cancer cutaneous involvement at the time of diagnosis was present in 0.5% of patients while 0.4% of patients were diagnosed because of cutaneous metastases [3, 13]. More recently, Rendi and Damian reported that only 0.5% of colorectal patients present cutaneous metastasis at the time of their initial diagnosis [14]. These authors found that cutaneous metastatic involvement was the first sign of the previously non-evident malignancy in only 0.8% of all cancer patients (367 cases). Therefore, cutaneous metastasis is rarely a sign of initial presentation of internal malignancy and often serves as an indicator of widespread metastatic disease with poor prognosis [7]. Nevertheless, a few cases of cutaneous metastases from colon cancer have been reported in the absence of liver involvement [15-17]. In patients with stage IV cancer, as our patient, the cancers most likely to appear on the skin in descending order of frequency include: breast, lung, kidney, colon, and stomach [12].

Clinically, the metastatic lesions do not have a uniformly characteristic appearance and they are often misdiagnosed mimicking simple cysts or benign connective tissue lesions such as pyogenic granuloma, granular cell tumor, a benign cyst, and benign subcutaneous nodule [18]. Therefore, atypical or persistent nodular lesions in patients with a history of systemic malignancy should be considered for biopsy to rule out metastasis [11]. Cutaneous involvement of colorectal carcinoma seems to favor sites of previous incisions, as a colostomy site, but sometimes the skin of scalp or face may be affected, and in these cases, the nodules have cystic appearance. In rare instances, cutaneous metastases from colorectal carcinoma show other appearances, including erysipelas-like lesions, alopecia neoplastica, and zosteriform pattern [19-22]. Alopecia neoplastica is a rare presentation of skin metastasis as single or multiple areas of cicatricial alopecia and is almost a unique presentation of metastasis from breast cancer [23].

Most metastatic adenocarcinomas from colon tumors can often be diagnosed or suspected based on histologic pattern alone resembling that of the primary tumor. In general, metastatic lobular breast carcinomas and moderately differentiated colonic carcinomas with typical tumor necrosis are among the easiest to recognize. However, there are difficult cases that require clinicopathologic correlation and immunohistochemical studies. When pathologists use only hematoxylin and eosin stain without clinical information, they can only correctly identify biopsied lesion specimens as metastatic in 66% of cases and can only further identify the primary origin in 44% of cases [18]. A positive staining with CEA is highly sensitive for colon tumors but not specific as this pattern also can be seen with breast, liver, and lung carcinomas. A combined panel of CK20, CEA, mucin, CDX2 for colonic carcinomas will be helpful in this differentiation [19]. Thus, using these staining patterns in combination with a positive CEA staining pattern may be helpful in diagnosis of metastatic adenocarcinomas from colon tumors, when the primary cancer is unknown [14].

Several different pathways are thought to be important for the mechanism of cutaneous metastasis. These include hematogenous and lymphatic spread, direct contiguous tissue invasion, and iatrogenic implantation [19, 24]. Lymphatic and vascular routes are the most common pathways, although differentiating the routes is difficult because they are interconnected. Lymphatic spread is the most common pathway for the initial spread of carcinoma. This mechanism for metastasis can be viewed as a sequence of steps, including vessel formation (angiogenesis), cell attachment, invasion (matrix degradation and cell motility), and cell proliferation [5, 19, 25].

Treatment almost exclusively is aimed at improving the patient’s quality of life. Given the poor mean survival time, excision and removal of metastases may be warranted to enhance the patient’s quality of life minimizing social embarrassment. Simple excision is usually the treatment of choice. Wound care for ulcerated lesions should also be provided because of the risk of infection.

Skin metastasis from colorectal cancer has to be considered as a poor prognostic sign due to widespread disease with median survival of 4.4 months, although an average of 18-month survival in 18 patients has also been reported in the literature [13, 26].

In conclusion, even though cutaneous metastases from colorectal carcinoma are rare, a high index of suspicion is necessary for early detection as they may remain unnoticed for a long period. Diagnostic biopsy is essential as they indicate extensive disease and poor prognosis. Recognition of cutaneous metastasis can help both the clinician and patient, by means of appropriate management of the therapeutics plans due to more accurate staging of the disease.

## References

[1] Hatoum HA, Abi Saad GS, Otrock ZK, Barada KA, Shamseddine AI (2011). Metastasis of colorectal carcinoma to the testes: clinical presentation and possible pathways. Int J Clin Oncol.

[2] Gupta SS, Singh O (2010). Carcinoma colon presenting as cutaneous metastasis to an old operative scar of hysterectomy. J Cancer Res Ther.

[3] Lookingbill DP, Spangler N, Sexton FM (1990). Skin involvement as the presenting sign of internal carcinoma. A retrospective study of 7316 cancer patients. J Am Acad Dermatol.

[4] Hu SC, Chen GS, Lu YW, Wu CS, Lan CC (2008). Cutaneous metastases from different internal malignancies: a clinical and prognostic appraisal. J Eur Acad Dermatol Venereol.

[5] Brownstein MH, Helwig EB (1972). Metastatic tumors of the skin. Cancer.

[6] Kauffman CL, Sina B (1997). Metastatic inflammatory carcinoma of the rectum: tumor spread by three routes. Am J Dermatopathol.

[7] Sarid D, Wigler N, Gutkin Z, Merimsky O, Leider-Trejo L, Ron IG (2004). Cutaneous and subcutaneous metastases of rectal cancer. Int J Clin Oncol.

[8] Balta AZ, Sucullu I, Ozdemir Y (2013). A rare clinical manifestation of rectal adenocarcinoma and synchronous scalp metastasis: A case report. Ulusal Cer Derg.

[9] Scheinfeld N (2006). Review of scalp alopecia due to a clinically unapparent or minimally apparent neoplasm (SACUMAN). Acta Derm Venereol.

[10] Gul U, Kilic A, Gonul M, Kulcu Cakmak S, Erinckan C (2007). Spectrum of cutaneous metastases in 1287 cases of internal malignancies: a study from Turkey. Acta Derm Venereol.

[11] Rogers JE, Ohinata A, Dasari A, Eng C (2014). Atypical metastatic presentations in colorectal cancer: a case series. Clin Colorectal Cancer.

[12] Wong CY, Helm MA, Helm TN, Zeitouni N (2014). Patterns of skin metastases: a review of 25 years' experience at a single cancer center. Int J Dermatol.

[13] Lookingbill DP, Spangler N, Helm KF (1993). Cutaneous metastases in patients with metastatic carcinoma: a retrospective study of 4020 patients. J Am Acad Dermatol.

[14] Rendi MH, Dhar AD (2003). Cutaneous metastasis of rectal adenocarcinoma. Dermatol Nurs.

[15] Wright PK, Jha MK, Barrett PD, Bain IM (2003). Colonic adenocarcinoma presenting as a cutaneous metastasis in an old operative scar. J Postgrad Med.

[16] Iwase K, Takenaka H, Oshima S, Kurozumi K, Nishimura Y, Yoshidome K, Tanaka T (1993). The solitary cutaneous metastasis of asymptomatic colon cancer to an operative scar. Surg Today.

[17] Morton BA, Scholes J, Kral JG (1986). An unusual presentation of colon cancer. J Surg Oncol.

[18] Sariya D, Ruth K, Adams-McDonnell R, Cusack C, Xu X, Elenitsas R, Seykora J (2007). Clinicopathologic correlation of cutaneous metastases: experience from a cancer center. Arch Dermatol.

[19] Alcaraz I, Cerroni L, Rutten A, Kutzner H, Requena L (2012). Cutaneous metastases from internal malignancies: a clinicopathologic and immunohistochemical review. Am J Dermatopathol.

[20] Tan KY, Ho KS, Lai JH, Lim JF, Ooi BS, Tang CL, Eu KW (2006). Cutaneous and subcutaneous metastases of adenocarcinoma of the colon and rectum. Ann Acad Med Singapore.

[21] Gul U, Kilic A, Akbas A, Aslan E, Demiriz M (2007). Alopecia neoplastica due to metastatic colon adenocarcinoma. Acta Derm Venereol.

[22] Damin DC, Lazzaron AR, Tarta C, Cartel A, Rosito MA (2003). Massive zosteriform cutaneous metastasis from rectal carcinoma. Tech Coloproctol.

[23] Kim HJ, Min HG, Lee ES (1999). Alopecia neoplastica in a patient with gastric carcinoma. Br J Dermatol.

[24] Hu SC, Chen GS, Wu CS, Chai CY, Chen WT, Lan CC (2009). Rates of cutaneous metastases from different internal malignancies: experience from a Taiwanese medical center. J Am Acad Dermatol.

[25] Royston D, Jackson DG (2009). Mechanisms of lymphatic metastasis in human colorectal adenocarcinoma. J Pathol.

[26] Schoenlaub P, Sarraux A, Grosshans E, Heid E, Cribier B (2001). [Survival after cutaneous metastasis: a study of 200 cases]. Ann Dermatol Venereol.

